# Social determinants of nonmedical sedative and opioid use among U.S. Latinos

**DOI:** 10.1371/journal.pone.0354367

**Published:** 2026-08-11

**Authors:** Patria Rojas, Weize Wang, Yajaira A. Cabrera Tineo, Said S. Salehe, Delaram Ghanooni, Mariana Sanchez

**Affiliations:** 1 Department of Health Promotion and Disease Prevention, Robert Stempel College of Public Health and Social Work, Florida International University, Miami, United States of America; 2 CRUSADA, Robert Stempel College of Public Health and Social Work, Florida International University, Miami, United States of America; 3 Department of Biostatistics, Robert Stempel College of Public Health and Social Work, Florida International University, Miami, United States of America; 4 Department of Environmental and Global Health, University of Florida, Gainesville, United States of America; 5 Department of Environmental Health Science, Robert Stempel College of Public Health and Social Work, Florida International University, Miami, United States of America; 6 Health Disparities Initiative, Robert Stempel College of Public Health and Social Work; Florida International University, Miami, United States of America; 7 Population Health Initiative, Robert Stempel College of Public Health and Social Work; Florida International University, Miami, United States of America; Virginia Commonwealth University School of Medicine, UNITED STATES OF AMERICA

## Abstract

The non-medical use of sedatives and prescription opioids is a growing public health concern, particularly among Latino populations. This study aim was to identify individual and social determinants associated with non-medical use of sedatives and prescription opioids among Latinos in the United States. A cross-sectional analysis was conducted using the National Institute of Health All of Us Dataset. The sample included 22,457 Hispanic/Latino adults with complete data. The main outcomes were lifetime street opioids use, and past 3-month non-medical use of sedatives and prescription opioids. Independent variables included depression, discrimination, education, income, nativity, and language spoken at home. The non-medical use of sedatives and opioids in the past 3 months was low (0.4% sedatives, 0.2% opioids). Older age, being male, and reporting depression, discrimination, and neighborhood disorder were associated with increased odds of opioids use. Higher education, income, foreign-born status, and non-English language spoken at home were protective factors. Findings highlight the need for culturally tailored interventions targeting high-risk adult Latino subgroups to address opioid and non-medical use of sedatives disparities.

## Introduction

Opioid Use Disorder (OUD) and its adverse outcomes represent a profound public health crisis in the United States of America (U.S.A). According to the National Institute on Drug Abuse (NIDA), approximately 105,000 people died from drug overdoses in 2022 [1]. Of these, 79,358 deaths – nearly 76% - involved opioids. The scale of the epidemic has expanded, with opioid-related overdose deaths increasing by almost 900% from 1999 to 2023. Since 2013, this surge has been overwhelmingly driven by the “third wave” of the crisis: synthetic opioids. In 2023 alone, 73,000 deaths were attributed to fentanyl, accounting for 69% of all drug overdose deaths [2]. This crisis has had devastating effects on national health and longevity. In 2022, synthetic opioid-related deaths were estimated to reduce U.S. life expectancy by 0.67 years, an impact comparable to, or exceeding, that of major chronic diseases [3]^.^ Beyond mortality, survivors of opioid overdose and those with non-fatal OUD face long-term neurological changes [4,5], increased susceptibility to infections [6] and cardiovascular complications [7,8].

While these impacts are nationwide, the burden is not shared equally. Emerging evidence indicates that the opioid crisis disproportionately affects racial and ethnic minority populations [9–11], with widening disparities in overdose death rates. These disparities are exacerbated by systemic social and structural inequities regarding risk reduction, prevention, and access to care [12]. Specifically, older Latinos —face unique vulnerabilities. Although prescription-specific research on Latinos remains limited, evidence suggests that the misuse of prescription pain relievers has contributed to rising rates of OUD among Latinos [13–15]. Recent studies indicate that Latino men experience unique patterns of substance use risk which are driven in part by low treatment utilization, nativity, and social stigma [16,17]. For Latino communities, factors influencing both risk and resilience are deeply rooted in their social context. A scoping review of the literature found that across the lifespan, neighborhood instability, age, and peer environment influenced substance abuse escalation among Latinos. Additionally, findings revealed that stigma and lack of social support hindered treatment and recovery among adult Latinos [18]. Socioeconomic status, language barriers, immigration status, and neighborhood conditions are social determinants of health (SDOH) that shape individuals’ health susceptibility and access to care [19]. However, institutional and societal biases have historically influenced the framing of opioid use in public discourse, often leaving Latino communities underrepresented in research and underserved in prevention efforts [15,20]. Poor employment opportunities, health system navigation challenges and low perception of treatment needs among Latinos are individual SDOH that consistently shape risk exposure, treatment access and outcomes of OUD [21]. The All of Us Research Program operationalizes SDOH across multiple domains including built environment, education and economic stability and its multidimensional framework is particularly well suited to examine health inequities and socio-structural drivers of OUD among Latinos.

Opioid related morbidity and mortality among Latinos have increased substantially in recent years underscoring the need of identifying its upstream determinants [22]. Although previous research have demonstrated that factors such as nativity, sex and ethnicity are significantly associated with opioid use and OUD [23], the role of SDOH in shaping OUD patterns among Latinos remains insufficiently examined [17,22]. Accordingly, the present study represents one of the first large scale investigation of opioid use among Latinos using a nationally representative sample from NIH All of US Research program (AoUs). The AoUs is a large-scale, nationally representative cohort designed to reflect the diversity of the U.S. population [24]. The current study aimed to identify key individual and social determinants of health associated with the non-medical use of sedatives and prescription opioids among Latinos in the U.S. Leveraging the large data from the NIH All of Us Research Program [24], we examined how factors such as depression, discrimination, educational attainment, income, and nativity status influence substance use patterns in this population.

## Materials and methods

**Fig 1:**
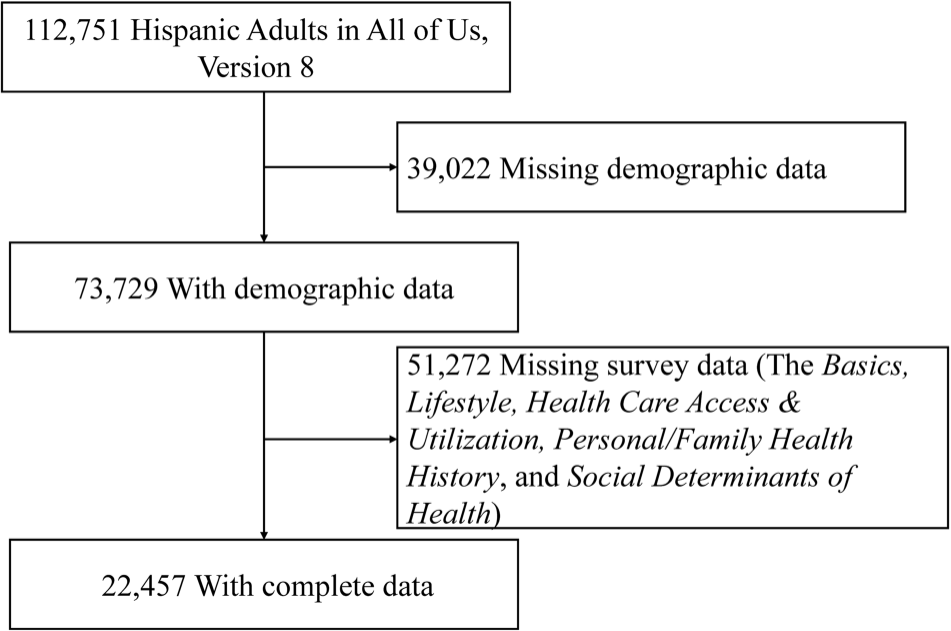
Schematic Flow Diagram for Sample Acquisition and Selection. Illustrates the stepwise exclusion process used to define the final study cohort. From an initial population of 112,751 Hispanic/Latino adults identified in the All of Us Research Program (Version 8), 39,022 individuals were excluded due to missing demographic information. Of the remaining 73,729 participants with available demographic data, an additional 51,272 were excluded for missing critical survey responses across five key modules: The Basics, Lifestyle, Health Care Access & Utilization, Personal/Family Health History, and Social Determinants of Health. This selection process resulted in a final analytical sample of 22,457 participants with complete data.

A retrospective study was conducted using the NIH AoUs Dataset. The analytic sample included only adult participants who self-identified as Hispanic/Latino. Of the initial 112,751 participants, 22,457 (20%) had completed the SDOH measure, including 13,968 U.S.-born and 8,489 foreign-born individuals (Fig 1). Methods and results of the present study are reported following the Strengthening the Reporting of Observational Studies in Epidemiology (STROBE) Statement for cross-sectional studies [25]. The subsample of 22,457 includes 13,968 U.S.-born and 8,489 foreign-born participants.

### Variables

Dependent variables included lifetime street opioids use, and past 3-month non-medical use of sedatives and prescription opioids. Lifetime street opioids use was selected as the outcome variable because no street opioids use was reported in the past three months. Given the secondary data analysis design of the current study, this represented the only feasible analytic option. Independent variables included demographic characteristics (age, gender, education, income, nativity, language spoken at home), mental health (depression), and social determinants (discrimination, neighborhood disorder).

### Demographics

Age in years was calculated using the difference between the survey date and the participant’s date of birth. Sex at birth was a dichotomous variable with male/female responses. Speaking a language other than English at home was a dichotomous variable with yes/no responses. Income was originally measured as annual household income, with 10 levels ranging from “less 10k” to “more 200k”. For the current study, we collapsed income into three categories: “ < $35k,” “$35k - $75k,” and “ > $75k.” Education was also recoded into three categories: “High School/GED or Less,” “Training After High School,” “College or Above.” Marital status was categorized into six levels in the AoUs data; we re-coded it to a binary variable with the levels “Married or in a relationship” and “Single, separated, or widowed.” Nativity was recoded into a dummy variable: US born or foreign-born.

### Social determinants of health measures

#### Social support.

The RAND MOS Social Support Survey Instrument includes 8 items rated on a 5-point Likert-type scale (1 = None of the time to 5 = All of the time) [26]. The total social support score was calculated as the means of all items. A higher score suggests a higher level of social support. The Cronbach’s alpha was 0.95 for both foreign- and U.S.-born Latinos.

#### Social cohesion.

The Social Cohesion Scale consists of 4 items rated on a 5-point Likert-type scale (1 = strongly agree to 5 = strongly disagree) [27]. The scale score was calculated as the mean of the four items. A higher scale score indicates a higher level of neighborhood cohesion. The Cronbach’s alpha was 0.86 for foreign-born Latinos and 0.87 for U.S.-born Latinos.

#### Loneliness.

The UCLA Loneliness Scale has 8 items rated on a 4-point Likert-type scale (1 = Never to 4 = Often) [28]. The summary score was the mean of all items. A higher score suggests a higher level of loneliness. The Cronbach’s alpha was 0.83 for foreign-born Latinos and 0.86 for U.S.-born Latinos.

#### Discrimination.

The Discrimination Scale includes 9 items rated on a 6-point Likert-type scale (0 = Never to 5 = Almost every day) [29]. The summary scale score was calculated using the mean of all the items. A higher score indicates a higher frequency of experiencing discrimination. The Cronbach’s alpha was 0.90 for foreign-born Latinos and 0.92 for U.S.-born Latinos.

#### Perceived stress.

The Perceived Stress Scale consists of 10 items rated on a 5-point Likert-type scale (1 = Never to 5 = Very Often) [30]. The scale score was calculated by using the mean of all the items. A higher score indicates a higher level of stress. The Cronbach’s alpha was 0.86 for foreign-born Latinos and 0.89 for U.S.-born Latinos.

#### Neighborhood disorder/safety.

The Neighborhood Disorder Scale includes 13 items rated on a 4-point Likert-type scale (1 = strongly disagree to 4 = strongly agree) [31]. Four items were reverse-scored. The summary score was calculated as the average of the items. A higher score indicates a poorer neighborhood safety/environment. The Cronbach’s alpha was 0.91 for foreign-born Latinos and 0.92 for U.S.-born Latinos.

#### Discrimination at healthcare settings.

The Discrimination at Healthcare Settings scale consists of 7 items rated on a 5-point Likert-type scale (1 = Never to 5 = Always) [32]. The summary score was computed as the average of the items. A higher score indicates a higher frequency of experiencing discrimination in healthcare settings. The Cronbach’s alpha was 0.89 for foreign-born Latinos and 0.90 for U.S.-born Latinos.

#### Religiosity/spirituality.

The Religiousness/Spirituality scale includes 6 items rated on a 6-point Likert typed scale (1 = Never or almost never to 6 = Many times a day) [33]. The scale score was calculated using the mean of all items. A higher scale score suggests a higher level of religiosity. The Cronbach’s alpha was 0.90 for foreign-born Latinos and 0.92 for U.S.-born Latinos.

#### Depression.

Lifetime self-reported depression was a recoded binary variable with “yes/no” responses. It was measured using the survey question “Have you ever been diagnosed with the following mental health or substance use conditions?” If the choice of “Depression” was selected, depression was recoded as “yes”.

### Non-medical drug use

#### Lifetime street opioids use.

The outcome variable, lifetime street opioids use, was recoded as a binary variable with “yes/no” responses. It was measured using self-reported responses to the survey question “In your LIFETIME, which of the following substances have you ever used (heroin, opium, etc.)?”. Lifetime street opioids use was recoded as “yes” if the choice “Which Drugs Used: Street Opioids Use” was selected.

#### Sedative use in the past three months.

The outcome variable, sedative use in the past three months, was defined as a binary variable. It was measured using self-reported responses to the survey question “In the past three months, how often have you used sedatives or sleeping pills for non-medical reasons (Valium, Serepax, Ativan, Xanax, Librium, Rohypnol, GHB, etc.)?”. Responses were recoded as ‘yes’ if participants reported use of “once or twice” or any more frequently choice in the original response options.

#### Prescription opioids use in the past three months.

The outcome variable, prescription opioid use in the past three months, was recorded as binary with “yes/no” responses. It was measured using self-reported responses to the survey question “In the past three months, how often have you used prescription opioids for non-medical reasons (fentanyl, oxycodone (OxyContin, Percocet), hydrocodone (Vicodin), methadone, buprenorphine, etc.)”. Sedative use in the past three months was recoded as “yes” if the frequency of use was “Once or Twice” or higher was selected.

### Statistical analysis methods

Descriptive analyses were conducted to summarize the study sample. For continuous variables, we calculated means and standard deviations, while categorical variables were summarized with frequencies and percentages. Overall sample characteristics, as well as stratified comparisons between participants included in the final regression analyses (complete cases), and those excluded due to missing values, were examined for group differences. Group differences were assessed using two sample t-tests for continuous variables and Chi-square tests for categorical variables. Because the social determinant of health (SDOH) questionnaire was optional and resulted in substantial missingness, the stratified comparison was used to evaluate potential differences in the demographic characteristics and social factors associated by missingness.

We employed logistic regression for the three dependent variables, respectively, including lifetime street opioid use, sedative use in the past three months, and prescription opioid use in the past three months. Due to the low event rate for the sedative and prescription opioid use in the past three months, Firth's penalized logistic regression was applied for the two outcomes. Independent variables included in the logistic models were depression, SDOH variables (social support, social cohesion, loneliness, discrimination, stress, neighborhood disorder/safety, discrimination in healthcare settings, religiosity/spirituality), and demographic variables (age, sex at birth, language spoken at home, education, annual income, and marital status). We estimated adjusted odds ratios with corresponding 95% confidence intervals based on the results from each model.

To evaluate potential selection bias due to the optional administration of social determinant of health (SDOH) questionnaire, inverse probability weighting (IPW) was applied. Questionnaire completion was modeled using multivariable logistic regression including demographic characteristics (age, sex at birth, education, income, language, nativity, marital status, and insurance), self-reported depression, and the three substance use outcomes. Stabilized inverse probability weights were derived from the predicted probability of questionnaire completion and truncated at the 99th percentile to reduce the influence of extreme values. Weighted logistic regression models with a quasibinomial link were then used to estimate adjusted odds ratios (aORs) and 95% confidence intervals. IPW analyses were conducted as sensitivity analyses to account for potential selection bias arising from non-random questionnaire completion. A p-value of less than 0.05 was considered statistically significant. All analyses were performed using the R programming environment (Version. 4.5.2) [34].

## Results

### Descriptive statistics of the sample

The sample included in the regression analyses were 22,457 Latino adults with a mean age of 46 years (SD = 15), and 8,489 (38%) foreign-born (Table 1). Among participants, 31% were male, and more than half (64%) reported speaking a language other than English at home. Nearly half (47%) had a college education or higher, and 39% reported an annual income of $35,000 or less. About 44% of the participants were single, separated, or widowed. Most participants (92%) had health insurance. The non-medical use of sedatives was 8.6% lifetime and 0.4% in the past three months, respectively. Non-medical use of prescription opioids was 8.0% lifetime and 0.2% in the past three months. About 1.9% of the participants reported ever using street opioids in their lifetime. No participant reported street opioid use in the past three months. Additionally, about 25% of participants reported depression.

**Table 1:** Baseline Characteristics of Participants Included and Excluded from Regression Analyses Due to Missing Data.

Variable	n (%) or mean (SD)			p
	Overall	Included in Regression Models		
		No	Yes	
N	112751	90294	22457	
Sex at birth: Male (%)	37004 (33.1)	30164 (33.7)	6840 (30.5)	<0.001
Race (%)				<0.001
American Indian or Alaska Native	2356 (2.1)	1761 (2.0)	595 (2.6)	
Asian	574 (0.5)	420 (0.5)	154 (0.7)	
Black or African American	1993 (1.8)	1602 (1.8)	391 (1.7)	
Middle Eastern or North African	221 (0.2)	159 (0.2)	62 (0.3)	
More than one population	5215 (4.6)	3683 (4.1)	1532 (6.8)	
Native Hawaiian or Other Pacific Islander	93 (0.1)	78 (0.1)	15 (0.1)	
None Indicated	91606 (81.2)	75956 (84.1)	15650 (69.7)	
White	10693 (9.5)	6635 (7.3)	4058 (18.1)	
Speak a language other than English at home: Yes (%)	19839 (66.7)	5440 (74.7)	14399 (64.1)	<0.001
Education (%)				<0.001
High School/GED or Less	49312 (45.1)	44131 (50.8)	5181 (23.1)	
Training After High School	29120 (26.6)	22368 (25.8)	6752 (30.1)	
College or Above	30839 (28.2)	20315 (23.4)	10524 (46.9)	
Income (%)				<0.001
<35k	41422 (53.9)	32574 (59.8)	8848 (39.4)	
35k-75k	18920 (24.6)	12530 (23.0)	6390 (28.5)	
> 75k	16579 (21.6)	9360 (17.2)	7219 (32.1)	
Marital status: Single, separated, or widowed (%)	53823 (49.4)	44058 (50.9)	9765 (43.5)	<0.001
Foreign Born (%)	51467 (45.9)	42978 (47.9)	8489 (37.8)	<0.001
Sedative use for non-medical reasons in the past 3 months (%)	331 (0.3)	233 (0.3)	98 (0.4)	<0.001
Prescription opioids use for non-medical reasons in the past 3 months (%)	221 (0.2)	186 (0.2)	35 (0.2)	0.151
Street opioids use in the past 3 months (%)	54 (0.001)	54 (0.1)	0 (0.0)	<0.001
Lifetime street opioids use (%)	2994 (2.7)	2569 (2.8)	425 (1.9)	<0.001
Self reported depression (%)	9988 (8.9)	4290 (4.8)	5698 (25.4)	<0.001
Have health insurance (%)	94784 (87.5)	74101 (86.3)	20683 (92.1)	<0.001
Age in years (mean (SD))	44.77 (15.93)	44.46 (16.04)	46.04 (15.43)	<0.001
Social support (mean (SD))	3.75 (1.09)	3.62 (1.14)	3.80 (1.07)	<0.001
Social cohesion (mean (SD))	3.55 (0.78)	3.51 (0.79)	3.57 (0.78)	<0.001
Loneliness (mean (SD))	1.91 (0.67)	1.90 (0.67)	1.92 (0.67)	0.005
Discrimination (mean (SD))	0.92 (0.96)	0.86 (0.99)	0.95 (0.95)	<0.001
Stress (mean (SD))	2.57 (0.78)	2.56 (0.74)	2.57 (0.79)	0.283
Neighborhood disorder/safety (mean (SD))	1.85 (0.57)	1.93 (0.56)	1.81 (0.57)	<0.001
Discrimination at healthcare settings (mean (SD))	1.61 (0.71)	1.55 (0.73)	1.62 (0.70)	<0.001
Religiosity/Spirituality (mean (SD))	3.90 (1.39)	4.14 (1.32)	3.82 (1.41)	<0.001

Note. Participants were excluded from the regression models that had missing values on one or more model variables. p-values are from chi-square tests (categorical variables) and two-sample t-tests (continuous variables) comparing participants included vs. excluded from the models.

By comparing the characteristics of participants included and excluded from regression analyses due to missing data (Table 1), participants reported significantly higher rates of sedative use for non-medical reasons in the past 3 months (0.4% vs. 0.3%, p < 0.001), street opioid use (1.9% vs. 2.8%, p < 0.001). However, the prescription opioid for non-medical reasons in the past 3 months did not differ significantly (p = 0.151). Beyond the outcomes, included participants differed significantly from excluded participants on most covariates, tending to be more likely to be White (18.1% vs. 7.3%), college-educated (46.9% vs. 23.4%), and higher in income (32.1% vs. 17.2% earning > $75k). In addition, the included participants were more likely to report depression (25.4% vs. 4.8%) and hold health insurance (92.1% vs. 86.3%).

### Regression analysis results

Results from crude logistic regression models show that higher odds of lifetime street opioid use were observed among males (OR = 3.83, 95% CI = [3.55, 4.14], S1 Table), and those who were not in a partnered relationship (OR = 2.35, 95% CI = [2.17, 2.54]). Higher odds were also observed for depression (OR = 1.36, 95% CI = [1.21, 1.52]), loneliness (OR = 2.35, 95% CI = [2.08, 2.65]), discrimination (OR=1.63, 95% CI = [1.52, 1.75]), stress (OR =1.74, 95% CI = [1.56, 1.95]), neighborhood disorder/safety concerns (OR = 1.93, 95% CI = [1.67, 2.21]), and discrimination in healthcare settings (OR=1.65, 95% CI = [1.50, 1.81]). Lower odds were observed among older individuals (OR = 0.996, 95% CI = [0.990, 0.999]), participants with college education or above (OR = 0.25, 95% CI = [0.22, 0.28]), annual income > $75,000 (OR = 0.21, 95% CI = [0.18, 0.25]), those who spoke a language other than English at home (OR = 0.38, 95% CI = [0.32, 0.45]), foreign-born individuals (OR = 0.19, 95% CI = [0.17, 0.21]), those with health insurance (OR = 0.58, 95% CI = [0.38, 0.81]), greater social support (OR = 0.73, 95% CI = [0.68, 0.78]), greater social cohesion (OR = 0.66, 95% CI [0.60, 0.74]), and higher religiosity/spirituality (OR = 0.87, 95% CI = [0.82, 0.93]) (all p < 0.05).

For sedative use in the past 3 months, higher odds were associated with older age (OR = 1.04, 95% CI = [1.03, 1.04], S1 Table), college education or above (OR = 1.94, 95% CI = [1.51, 2.51]), foreign-born status (OR = 1.82, 95% CI = [1.46, 2.28]), health insurance coverage (OR = 1.73, 95% CI = [1.17, 2.68]), depression (OR = 1.72, 95% CI = [1.25, 2.32]), and religiosity/spirituality (OR = 1.19, 95% CI = [1.04, 1.36]). Lower odds were observed among males (OR = 0.78, 95% CI = [0.61, 0.99]), individuals with greater social support (OR = 0.85, 95% CI = [0.73, 1.00]), greater social cohesion (OR = 0.79, 95% CI = [0.63, 1.00]), and higher stress (OR = 0.78, 95% CI = [0.62, 0.98]) (all p < 0.05).

For prescribed opioid use in the past 3 months, lower odds were observed among males (OR = 0.50, 95% CI = [0.36, 0.69], S1 Table), participants with a college education or above (OR = 0.60, 95% CI = [0.41, 0.87]), annual income greater than $75,000 (OR = 0.38, 95% CI = [0.22, 0.61]), and those reporting greater social support (OR = 0.74, 95% CI = [0.59, 0.94]). Higher odds were associated with loneliness (OR = 1.59, 95% CI = [1.07, 2.33]), discrimination (OR = 1.33, 95% CI = [1.03, 1.67]), and stress (OR = 1.48, 95% CI = [1.05, 2.09]) (all p < 0.05).

Results from multivariable logistic models show that older age was consistently associated with higher odds of the three types of substance use: lifetime street opioid use (aOR = 1.01, 95% CI = [1.004, 1.02], Table 2), past three-month non-medical prescription sedative use (aOR = 1.03, 95% CI = [1.02, 1.05]), and past three-month non-medical opioid use (aOR = 1.03, 95% CI = [1.01, 1.05]). Males were more likely to report lifetime street opioid use (aOR = 2.39, 95% CI = [1.94, 2.93]) but had lower odds of recent prescription opioid use compared to females (aOR = 0.37, 95% CI = [0.12, 0.91]). Higher education (college or more, aOR = 0.41, 95% CI = [0.30, 0.55]) and higher income (aOR for 35k-75k = 0.65, 95% CI = [0.51, 0.84]; aOR for>75k = 0.53, 95% CI = [0.38, 0.72]) were protective against lifetime street opioid use, while those with training after high school had increased odds of recent prescription opioid use (aOR = 2.24, 95% CI = [1.01, 5.44]). Speaking a language other than English at home and being foreign-born were associated with lower odds of lifetime street opioid use (aOR = 0.50, 95% CI = [0.40, 0.61]) but associated with higher odds of recent sedative use (aOR = 1.80, 95% CI = [1.10, 3.03]).Depression (aOR = 1.74, 95% CI = [1.41, 2.16]), discrimination (aOR = 1.22, 95% CI = [1.08, 1.37]), and neighborhood disorder (aOR = 1.25, 95% CI = [1.03, 1.52]) significantly increased the odds of lifetime street opioid use, whereas loneliness (aOR = 1.65, 95% CI = [1.07, 2.51]) was associated with increased odds of sedative use in the past three months. Overall, demographic factors and SDOH played varying roles across different types of substance us

**Table 2:** Estimates from Multiple Logistic Regression for Lifetime Street Opioids Use, and Sedative Use and Prescribed Opioids Use in the Past 3 Months (N = 22,457).

Independent Variables	Lifetime Street Opioids Use		Sedative Use in the Past 3 Months		Prescribed Opioids Use in the Past 3 Months	
	aOR [95% Confidence Interval]	p-value	aOR [95% Confidence Interval]	p-value	aOR [95% Confidence Interval]	p-value
Age in years	**1.01 [1.004, 1.02]**	**0.002**	**1.03 [1.02, 1.05]**	**<0.0001**	**1.03 [1.01, 1.05]**	**0.040**
Sex at birth: Male vs. Female	**2.39 [1.94, 2.93]**	**<0.0001**	0.73 [0.45, 1.14]	0.169	**0.37 [0.12, 0.91]**	**0.029**
Education:						
Training After High School vs. High School/GED or Less	0.93 [0.74, 1.18]	0.542	0.94 [0.51, 1.73]	0.843	**2.24 [1.01, 5.44]**	**0.049**
College or Above vs. High School/GED or Less	**0.41 [0.3, 0.55]**	**<0.0001**	1.22 [0.7, 2.16]	0.487	0.69 [0.23, 2]	0.494
Income:						
35k-75k vs. < 35k	**0.65 [0.51, 0.84]**	**0.001**	1.35 [0.8, 2.29]	0.264	0.48 [0.18, 1.13]	0.094
> 75k vs. < 35k	**0.53 [0.38, 0.72]**	**0.000**	1.28 [0.71, 2.31]	0.415	0.52 [0.16, 1.46]	0.226
Speak a language other than English at home: Yes vs. No	**0.5 [0.4, 0.61]**	**<0.0001**	**1.8 [1.1, 3.03]**	**0.018**	1.38 [0.65, 3.1]	0.408
Foreign-Born vs. U.S.-Born	**0.31 [0.23, 0.43]**	**<0.0001**	1.26 [0.81, 1.97]	0.307	1.09 [0.5, 2.33]	0.826
Marital status:						
Single, divorced, separated, or widowed vs. Married or in a relationship	1.14 [0.92, 1.42]	0.235	0.89 [0.57, 1.38]	0.596	1.33 [0.65, 2.84]	0.442
Have health insurance	1.06 [0.72, 1.62]	0.772	0.87 [0.42, 2.07]	0.733	2.18 [0.56, 19.67]	0.306
Depression	**1.74 [1.41, 2.16]**	**<0.0001**	1.57 [0.97, 2.49]	0.068	1.59 [0.76, 3.26]	0.215
Social support	0.96 [0.86, 1.07]	0.460	0.91 [0.74, 1.14]	0.420	0.83 [0.58, 1.19]	0.305
Social cohesion	0.97 [0.84, 1.12]	0.673	1.34 [0.99, 1.83]	0.060	1.03 [0.63, 1.68]	0.909
Loneliness	1.22 [0.99, 1.5]	0.060	**1.65 [1.07, 2.51]**	**0.024**	1.06 [0.52, 2.14]	0.873
Discrimination	**1.22 [1.08, 1.37]**	**0.001**	0.79 [0.54, 1.11]	0.183	1.02 [0.65, 1.55]	0.940
Stress	1.02 [0.86, 1.2]	0.828	0.86 [0.61, 1.21]	0.381	1.06 [0.6, 1.88]	0.836
Neighborhood disorder/safety	**1.25 [1.03, 1.52]**	**0.025**	1.22 [0.8, 1.83]	0.353	1.15 [0.59, 2.19]	0.682
Discrimination at healthcare settings	1.1 [0.94, 1.29]	0.227	0.7 [0.43, 1.09]	0.116	1.2 [0.67, 1.98]	0.532
Religiosity/Spirituality	1.02 [0.95, 1.1]	0.600	1.04 [0.88, 1.23]	0.669	0.99 [0.75, 1.3]	0.930

Note. aOR = Adjusted Odds Ratio.

### Inverse probability weighting sensitivity analysis

Results from the inverse probability weighting analysis were highly consistent with those observed in the complete-case multivariable logistic regression models (Table 3). The direction, magnitude, and statistical significance of the associations between the demographic characteristics, SDOH factors, and the three substance use outcomes remained largely unchanged after weighting. These findings suggest that the primary results were robust to potential bias arising from missing SDOH data and support the validity of the complete-case analyses.

**Table 3:** Inverse Probability Weighting Logistic Regression Estimates for Lifetime Street Opioids Use, and Sedative Use and Prescribed Opioids Use in the Past 3 Months.

Independent Variables	Lifetime Street Opioids Use		Sedative Use in the Past 3 Months		Prescribed Opioids Use in the Past 3 Months	
	aOR [95% Confidence Interval]	p-value	aOR [95% Confidence Interval]	p-value	aOR [95% Confidence Interval]	p-value
Age in years	**1.01 [1.01, 1.02]**	**<0.001**	**1.03 [1.02, 1.05]**	**<0.001**	**1.03 [1.001, 1.05]**	**0.031**
Sex at birth: Male vs. Female	**2.39 [1.95, 2.94]**	**<0.001**	0.72 [0.45, 1.13]	0.153	**0.33 [0.11, 0.97]**	**0.044**
Education:						
Training After High School vs. High School/GED or Less	0.93 [0.74, 1.18]	0.563	0.95 [0.5, 1.82]	0.885	**2.34 [1.001, 5.47]**	**0.049**
College or Above vs. High School/GED or Less	**0.41 [0.3, 0.56]**	**<0.001**	1.25 [0.66, 2.39]	0.496	0.69 [0.24, 2.01]	0.499
Income:						
35k-75k vs. < 35k	**0.66 [0.51, 0.84]**	**0.001**	1.36 [0.78, 2.35]	0.277	0.45 [0.18, 1.13]	0.090
> 75k vs. < 35k	**0.53 [0.38, 0.73]**	**<0.001**	1.29 [0.67, 2.5]	0.446	0.49 [0.16, 1.51]	0.215
Speak a language other than English at home: Yes vs. No	**0.5 [0.4, 0.61]**	**<0.001**	**1.82 [1.05, 3.15]**	**0.032**	1.42 [0.67, 2.97]	0.358
Foreign-Born vs.U.S.-Born	**0.31 [0.22, 0.44]**	**<0.001**	1.26 [0.76, 2.1]	0.368	1.08 [0.48, 2.44]	0.851
Marital status:						
Single, divorced, separated, or widowed vs. Married or in a relationship	1.14 [0.91, 1.44]	0.260	0.88 [0.55, 1.42]	0.611	1.34 [0.6, 3.03]	0.475
Have health insurance	1.06 [0.71, 1.59]	0.768	0.92 [0.42, 2.03]	0.839	3.25 [0.39, 26.85]	0.275
Depression	**1.75 [1.4, 2.18]**	**<0.001**	1.57 [0.97, 2.54]	0.066	1.59 [0.77, 3.27]	0.206
Social support	0.96 [0.86, 1.07]	0.478	0.92 [0.73, 1.15]	0.446	0.83 [0.57, 1.22]	0.351
Social cohesion	0.97 [0.83, 1.13]	0.692	1.34 [0.96, 1.88]	0.087	1.03 [0.59, 1.81]	0.916
Loneliness	1.22 [0.98, 1.52]	0.071	**1.64 [1.04, 2.6]**	**0.033**	1.07 [0.47, 2.44]	0.877
Discrimination	**1.22 [1.09, 1.37]**	**0.001**	0.78 [0.52, 1.17]	0.236	1.02 [0.71, 1.45]	0.931
Stress	1.02 [0.85, 1.22]	0.835	0.86 [0.63, 1.17]	0.332	1.06 [0.59, 1.91]	0.841
Neighborhood disorder/safety	**1.25 [1.02, 1.53]**	**0.029**	1.21 [0.78, 1.88]	0.391	1.14 [0.5, 2.55]	0.759
Discrimination at healthcare settings	1.1 [0.94, 1.29]	0.233	0.69 [0.36, 1.3]	0.248	1.16 [0.75, 1.77]	0.506
Religiosity/Spirituality	1.02 [0.95, 1.1]	0.601	1.04 [0.87, 1.24]	0.692	0.99 [0.74, 1.32]	0.938

Note. aOR = Adjusted \Odds Ratio.

## Discussion

The present study sought to identify key individual and social determinants of health associated with the non-medical use of sedatives and prescription opioids among Latinos in the U.S. using a large nationally representative sample. Current findings underscore significant associations between individual characteristics, social determinants, and opioid use behaviors among U.S. Latinos. Specifically, we found higher education, income, foreign-born status, and non-English language use at home were associated with lower odds of opioid misuse, suggesting these cultural and structural contexts may buffer against maladaptive opioid use. Conversely, we found mental health challenges and adverse neighborhood conditions, such as neighborhood disorder, elevated risks for opioid misuse.

Current findings on the links between discrimination, income, neighborhood disorder, and lifetime opioid use align with broader literature emphasizing the role of stigma, discrimination, and socioeconomic instability in substance use outcomes [35]. For example, institutional discrimination and poor infrastructural context have historically shaped access to treatment and public perceptions of opioid use, often marginalizing communities of color. Moreover, adverse SDOH such as housing instability and food insecurity have been shown to reduce medication adherence and exacerbate chronic health conditions [36–39]. These individual and structural factors are, therefore, particularly important for prevention efforts and treatment strategies addressing opioid use. Conversely, our data shows that foreign-born status and non-English language use at home are associated with lower odds of lifetime street opioid use. This observation supports the findings in the broader literature about the “immigrant-paradox,” where recent immigrants often exhibit better health outcomes than their U.S.-born counterparts despite lower socioeconomic status [40–42]. These cultural contexts may serve as a buffer against substance use through different social norms regarding drug use.

The consistent link between older age and increased odds of non-medical use of sedatives and prescription opioids is a critical public health finding. Specifically, older age is associated with physiological changes that influence individuals’ metabolism and bodily response to opioids [43]. With aging, opioid use also converges with other age-related risk factors and predisposes older Latinos to a syndemic of morbidities, such as HIV/AIDS and other chronic conditions [44]. Because older Latinos represent a growing demographic segment, there is an urgent need to address the unique vulnerabilities they face, which requires careful consideration of their socio-cultural context in the development of effective culturally tailored interventions addressing opioid use.

The marked sex-based divergence—where males had over double the odds of lifetime street opioid use (aOR = 2.39), but significantly lower odds of recent prescription opioid use (aOR = 0.37) compared to females—may suggests distinct gender pathways to substance misuse. These findings imply that Latino men may face greater barriers to formal pain management or are more susceptible to the illicit ‘street’ drug market, whereas Latino women may be more at risk for the transition from medical prescription to non-medical use. The possible reasons for this divergence may include men being more risk-taking than women, overrepresented in high-risk occupations, and being more likely to sustain injuries requiring opioid use or illegal substance use whereas women’s recent opioid use may be due to increased community access to prescription opioids. This sex divergence suggests that prevention and intervention strategies must be gender-informed; outreach for men might focus on harm reduction and the risks of illicit street opioids, whereas clinical screenings for women should prioritize the monitoring of prescription medication adherence and potential misuse.

The observed intersection of age and sex reveals distinct risk patterns, with higher odds of lifetime street opioid use among males suggest a need for gender responsive prevention strategies; while the higher likelihood of recent non-medical prescription opioid use among females underscores the importance of safer prescribing policies, and monitoring and pain management approaches tailored to women. Collectively, these results support the integration of age‑ and sex‑specific interventions into routine clinical care to address substance‑related harms across the life course. Tailored prevention strategies are needed to address the unique vulnerabilities and cultural strengths of Latino subgroups. Interventions should prioritize older, low-income, English-speaking Latinos who may be at elevated risk. Community-based approaches that integrate gender-informed and culturally competent care, mental health support, and harm reduction strategies—such as naloxone distribution and medication-assisted treatment—can help mitigate the impact of opioid misuse.

## Supporting information

S1 Table Estimates from Crude Logistic Regression for Lifetime Street Opioids Use, Sedative Use and Prescribed Opioids Use in the Past 3 Months. (DOCX)
