# Supplementary material for: Social determinants of nonmedical sedative and opioid use among U.S. Latinos
Source: PLoS One. 2026 Aug 11;21(8):e0354367. doi: 10.1371/journal.pone.0354367 (PMC13460567; doi:10.1371/journal.pone.0354367)
Supplement: Supplementary file 1 [file pone.0354367.s001.docx]

| **Support Table 1. Estimates from Crude Logistic Regression for Lifetime Street Opioids Use, Sedative Use and Prescribed Opioids Use in the Past 3 Months.** | | | | | | | | | | | | |
| --- | --- | --- | --- | --- | --- | --- | --- | --- | --- | --- | --- | --- |
| **Independent Variables** | **Lifetime Street Opioids Use** | | | | **Sedative Use in the Past 3 Months** | | | | **Prescribed Opioids Use in the Past 3 Months** | | | |
|  | **OR** | **95% CI** | | **p-value** | **OR** | **95% CI** | | **p-value** | **OR** | **95% CI** | | **p-value** |
|  |  | **LL** | **UL** |  |  | **LL** | **UL** |  |  | **LL** | **UL** |  |
| Age in years | **0.996** | **0.99** | **0.999** | **0.001** | **1.04** | **1.03** | **1.04** | **<0.001** | **1.02** | **1.01** | **1.02** | **0.000** |
| Sex at birth: Male vs. Female | **3.83** | **3.55** | **4.14** | **<0.001** | **0.78** | **0.61** | **0.99** | **0.043** | **0.50** | **0.36** | **0.69** | **0.000** |
| Education: |  |  |  |  |  |  |  |  |  |  |  |  |
| Training After High School vs. High School/GED or Less | **0.81** | **0.74** | **0.87** | **<0.001** | 1.21 | 0.90 | 1.62 | 0.203 | 1.27 | 0.94 | 1.71 | 0.123 |
| College or Above vs. High School/GED or Less | **0.25** | **0.22** | **0.28** | **<0.001** | **1.94** | **1.51** | **2.51** | **0.000** | **0.60** | **0.41** | **0.87** | **0.006** |
| Income: |  |  |  |  |  |  |  |  |  |  |  |  |
| 35k-75k vs. < 35k | **0.32** | **0.28** | **0.36** | **<0.001** | 1.00 | 0.73 | 1.35 | 0.983 | 0.88 | 0.62 | 1.23 | 0.463 |
| > 75k vs. < 35k | **0.21** | **0.18** | **0.25** | **<0.001** | 1.16 | 0.85 | 1.57 | 0.357 | **0.38** | **0.22** | **0.61** | **0.000** |
| Speak a language other than English at home: Yes vs. No | **0.38** | **0.32** | **0.45** | **<0.001** | **1.60** | **1.07** | **2.47** | **0.020** | 1.26 | 0.70 | 2.39 | 0.454 |
| Foreign-Born vs.U.S.-Born | **0.19** | **0.17** | **0.21** | **<0.001** | **1.82** | **1.46** | **2.28** | **0.000** | 0.79 | 0.60 | 1.03 | 0.086 |
| Marital status: |  |  |  |  |  |  |  |  |  |  |  |  |
| Single, divorced, separated, or widowed vs. Married or in a relationship | **2.35** | **2.17** | **2.54** | **<0.001** | 0.91 | 0.73 | 1.13 | 0.406 | 1.27 | 0.97 | 1.66 | 0.080 |
| Have health insurance | **1.58** | **1.38** | **1.81** | **<0.001** | **1.73** | **1.17** | **2.68** | **0.005** | **3.48** | **1.87** | **7.50** | **0.000** |
| Depression | **1.36** | **1.21** | **1.52** | **<0.001** | **1.72** | **1.25** | **2.32** | **0.001** | 1.34 | 0.86 | 1.98 | 0.185 |
| Social support | **0.73** | **0.68** | **0.78** | **<0.001** | **0.85** | **0.73** | **1.00** | **0.043** | **0.74** | **0.59** | **0.94** | **0.014** |
| Social cohesion | **0.66** | **0.60** | **0.74** | **<0.001** | **1.29** | **1.03** | **1.63** | **0.027** | 0.90 | 0.64 | 1.28 | 0.557 |
| Loneliness | **2.35** | **2.08** | **2.65** | **<0.001** | 1.07 | 0.82 | 1.38 | 0.614 | **1.59** | **1.07** | **2.33** | **0.021** |
| Discrimination | **1.63** | **1.52** | **1.75** | **<0.001** | **0.74** | **0.59** | **0.91** | **0.004** | **1.33** | **1.03** | **1.67** | **0.029** |
| Stress | **1.74** | **1.56** | **1.95** | **<0.001** | **0.78** | **0.62** | **0.98** | **0.034** | **1.48** | **1.05** | **2.09** | **0.028** |
| Neighborhood disorder/safety | **1.93** | **1.67** | **2.21** | **<0.001** | 0.85 | 0.62 | 1.16 | 0.314 | 1.58 | 1.00 | 2.45 | 0.053 |
| Discrimination at healthcare settings | **1.65** | **1.50** | **1.81** | **<0.001** | **0.66** | **0.48** | **0.88** | **0.004** | **1.69** | **1.24** | **2.23** | **0.001** |
| Religiosity/Spirituality | **0.87** | **0.82** | **0.93** | **<0.001** | **1.19** | **1.04** | **1.36** | **0.009** | 1.06 | 0.87 | 1.30 | 0.551 |

Note. OR = Odds Ratio.
